# Supplementary material for: How can past sea level be evaluated from traces of anthropogenic layers in ancient saltpans?
Source: PLoS One. 2023 Jul 19;18(7):e0287977. doi: 10.1371/journal.pone.0287977 (PMC10355413; doi:10.1371/journal.pone.0287977)
Supplement: Attachment — Submitted filename: Response to Reviewers.docx [file pone.0287977.s004.docx]

**Response to the editor's and reviewer's comments and concerns**

Dear Editor and Reviewers,	11.5.2023

Below please find all the answers to the editors and the reviewers’ comments and concerns.

our response is in *italic fonts*.

**Journal Requirements:**

1. When submitting your revision, we need you to address these additional requirements.

Please ensure that your manuscript meets PLOS ONE's style requirements, including those for file naming. The PLOS ONE style templates can be found at

https://journals.plos.org/plosone/s/file?id=wjVg/PLOSOne_formatting_sample_main_body.pdf and

https://journals.plos.org/plosone/s/file?id=ba62/PLOSOne_formatting_sample_title_authors_affiliations.pdf

***Answer:*** *We rechecked the manuscript, and it meets PLOS ONE style requirements.*

2. In your manuscript, please provide additional information regarding the specimens used in your study. Ensure that you have reported specimen numbers and complete repository information, including museum name and geographic location.

***Answer:*** *Although WoRMS, which we refer to in our manuscript, includes all up-to-date taxonomic information regarding species used in our study, we accepted the editor's request, and added, a taxonomic reference list of the most common species, in the supplementary data. The list was added to supplementary data 1 & 2 and we referred to it in the material and methods chapter. We added information about the location where the illustrated specimens are housed for potential future analyses in the Materials and Methods chapter (lines 228-229). We added ecological preference data to the supplementary tables, i.e., salinity-tolerance ranges of the relevant species.*

If permits were required, please ensure that you have provided details for all permits that were obtained, including the full name of the issuing authority, and add the following statement:

'All necessary permits were obtained for the described study, which complied with all relevant regulations.'

If no permits were required, please include the following statement:

'No permits were required for the described study, which complied with all relevant regulations.'

For more information on PLOS ONE's requirements for paleontology and archeology research, see <https://journals.plos.org/plosone/s/submission-guidelines#loc-paleontology-and-archaeology-research>.

***Answer:*** *No permits were required for the described study, which complied with all relevant regulations.*

3. Thank you for stating the following in the Acknowledgments Section of your manuscript:

"This study at the Department of Maritime Civilizations, School of Archaeology and Maritime Cultures, University of Haifa, was funded by a Sir Maurice and Lady Irene Hatter Research grant for Maritime Studies for which we are thankful. The research was partly funded through the Croatian Science Foundation grant agreement IP-04-2019-8505 QMAD. We thank the graphic artist Noga Yoselevich from the Department of Geography and Environmental Studies, University of Haifa, for the figures’ design. Special gratitude goes to Jona Petešić, associate for cultural heritage at the Nature Park Telašćica on Dugi Otok, whose experience and local knowledge greatly helped us while surveying the submerged saltpans of Brbinj. We would like to express our gratitude to Petar Crnčan from the Croatian Natural History Museum for his assistance in identifying the macro-fauna on the site."

We note that you have provided funding information that is not currently declared in your Funding Statement. However, funding information should not appear in the Acknowledgments section or other areas of your manuscript. We will only publish funding information present in the Funding Statement section of the online submission form. Please remove any funding-related text from the manuscript and let us know how you would like to update your Funding Statement. Currently, your Funding Statement reads as follows:

"The author(s) received no specific funding for this work."

Please include your amended statements within your cover letter; we will change the online submission form on your behalf**.**

***Answer:*** *Funding information was removed from the revised Acknowledgments, please correct our funding statement as follows: "This research was partly funded through the Croatian Science Foundation grant agreement IP-04-2019-8505 QMAD"*

4. We note that Figure 1 in your submission contain [map/satellite] images which may be copyrighted. All PLOS content is published under the Creative Commons Attribution License (CC BY 4.0), which means that the manuscript, images, and Supporting Information files will be freely available online, and any third party is permitted to access, download, copy, distribute, and use these materials in any way, even commercially, with proper attribution. For these reasons, we cannot publish previously copyrighted maps or satellite images created using proprietary data, such as Google software (Google Maps, Street View, and Earth). For more information, see our copyright guidelines: http://journals.plos.org/plosone/s/licenses-and-copyright**.**

We require you to either (a) present written permission from the copyright holder to publish these figures specifically under the CC BY 4.0 license, or (b) remove the figures from your submission:

a. You may seek permission from the original copyright holder of Figure(s) [#] to publish the content specifically under the CC BY 4.0 license.

We recommend that you contact the original copyright holder with the Content Permission Form (http://journals.plos.org/plosone/s/file?id=7c09/content-permission-form.pdf) and the following text:

“I request permission for the open-access journal PLOS ONE to publish XXX under the Creative Commons Attribution License (CCAL) CC BY 4.0 (http://creativecommons.org/licenses/by/4.0/). Please be aware that this license allows unrestricted use and distribution, even commercially, by third parties. Please reply and provide explicit written permission to publish XXX under a CC BY license and complete the attached form.”

Please upload the completed Content Permission Form or other proof of granted permissions as an "Other" file with your submission.

In the figure caption of the copyrighted figure, please include the following text: “Reprinted from [ref] under a CC BY license, with permission from [name of publisher], original copyright [original copyright year].”

b. If you are unable to obtain permission from the original copyright holder to publish these figures under the CC BY 4.0 license or if the copyright holder’s requirements are incompatible with the CC BY 4.0 license, please either i) remove the figure or ii) supply a replacement figure that complies with the CC BY 4.0 license. Please check copyright information on all replacement figures and update the figure caption with source information. If applicable, please specify in the figure caption text when a figure is similar but not identical to the original image and is therefore for illustrative purposes only.

The following resources for replacing copyrighted map figures may be helpful:

USGS National Map Viewer (public domain): http://viewer.nationalmap.gov/viewer/

The Gateway to Astronaut Photography of Earth (public domain): http://eol.jsc.nasa.gov/sseop/clickmap/

Maps at the CIA (public domain): <https://www.cia.gov/library/publications/the-world->factbook/index.html and https://www.cia.gov/library/publications/cia-maps-publications/index.html

NASA Earth Observatory (public domain): http://earthobservatory.nasa.gov/

Landsat: http://landsat.visibleearth.nasa.gov/

USGS EROS (Earth Resources Observatory and Science (EROS) Center) (public domain): http://eros.usgs.gov/#

Natural Earth (public domain): <http://www.naturalearthdata.com/>

***Answer:*** *Figure 1a-c was replaced, its source now is: "ESRI, Maxar, Earthstar geographics, and the GIS User Community". Figure 1d is a Digital Surface Model of the site, generated by the author and first published by Bechor et al. (2020). The figure caption has been changed in the revised manuscript accordingly.*

**Additional Editor Comments:**

I have now received the comments of three external reviewers raising major concerns on your Ms, particularly Reviewer 1 who rejected it. Reviewer 1 recognizes the value of the Ms but also stresses its limitations and how it can make a significant contribution to sea level studies. The reviewer suggests a re-focused of the Ms and provides some good points to do so. Reviewer 2 also acknowledges the outcomes but finds results not entirely conclusive and some parts unclear. Reviewer 3 highlights the potential of the Ms and considers it novel. The same reviewer suggests to provide a more robust paleoenvironmental interpretation of core units, to better constrain the ecology of species and include a part with the data limitation. On the basis of their comments, I am very sorry to not accept the Ms in the present form and reject the Ms but I strongly invite the authors to benefit of reviewers’ comments and encourage its resubmission.

***Answer:*** *This study* *is part* *of a larger project aiming to develop a new approach for obtaining past sea-level data using ancient intertidal saltpans as sea-level indicators, like the case of fishponds. Unlike fishponds which usually are limited to early Roman times, saltpans were used continuously for at least the last two millennia, spread around the Mediterranean Sea, and therefore have great potential as a sea-level tool. The archaeological arguments were explained in detail by Bechor et al. (2020). The current manuscript adds an additional discipline based on coring in the saltpans and analyzing its sediments. The study aims to identify the salt production unit, and date the beginning and ending of this period which was not feasible based on archaeology. The salt production layers and the ages provide lower sea level limiting points, which is considered important information for reconstructing past sea levels. The enquired data from the current study agrees with the previous study's data (see Fig 11).*

*We agree with reviewers 2 and 3 and added more detailed paleoenvironmental interpretations to the discussion chapter.*

**Reviewer #1**: Manuscript #: PONE-D-23-07685

Title: How can past sea level be evaluated from traces of anthropogenic layers in ancient saltpans?

Authors: Benny Bechor; Simona Avnaim-Katav; Steffen Mischke; Slobodan Miko; Ozren Hasan; Maja Grisonic; Irena Radić Rossi; Barak Herut; Nimer Taha; Naomi Porat; Dorit Sivan

Article type: Research Article

Dear Dr Frontalini,

Thank you so much for considering me to review this manuscript (details provided above). This manuscript reads quite well and was also very interesting. As and introduction, I work on coastal system and environmental changes on those systems using chronological, sedimentological, geochemical and micropaleontological proxies. This manuscript sounded really interesting to me, I must say. I would say that the research is very valuable and worth publishing but, and here is the “but”, I do not see how this work responds to the goal of using these anthropogenic remains for sea-level studies. Much less at the time scales that the authors identified. I am sorry to say but I struggle to see how this study make a significant contribution to sea level studies. Few notes on why I say this. The authors did not create sea level index points (SLIPs) and as in fact they mention limiting points.

***Answer:*** *As mentioned above, this study provides sea level lower limiting points with OSL dating which are important information for reconstructing past sea levels (Vacchi et al., 2016; Hijma et al., 2015).*

In fact, these locations need to be fully subtidal to allow tides to properly flood them and allow us to, more or less successfully, create a relationship between the depositional environment and the tidal frame. If they are only flooded partially, the problem we would encounter would be that the hydrodynamics are quite complex and we cannot reconstruct the changes in the tidal frame locally.

***Answer:*** *We describe in the Introduction chapter (lines 46-50) the relations between the tide level and the saltpans during the salt-production process. Lines 99-106 present the relations between the saltpan's indicative structures (separation wall and the sluice gates) and the tidal range. Thus, we argue that the saltpans are tightly connected to the contemporaneous sea level and can serve as good past sea-level indicators.*

At larger time scales, this could not be such a great problem but here we are talking about contributions for the last 1000 years. To add to this, the region has significant tectonic activity with vertical land movements that exceed 2 mm/yr locally (based on GPS data).

***Answer:*** *This subject is discussed in the manuscript in lines 546-555 where it is emphasized that unlike the southern part of the EAC, the center (the Dalmatian coast) is considered* *stable based on GPS measurements (Vacchi et al., 2016; Pace et al., 2022).*

Combining isostatic and tectonic vertical land movement makes it more complicated to deconvolve the factors controlling the relative sea-level changes, which, in turn, requires higher precision SLIPs. In all fairness, I kind of struggle to see how the hydrodynamics works here although I know that the authors refer to other works but that requires from the reader/reviewer to do our own research. To finalize this section, I think that the analysis of the tectonic activity and GIA data along with other SLIPs is quite brief and would need further detail (if that is the goal of the paper). For instance, are all the SLIPs in Fig 11 located with enough proximity to each other that vertical land movements would allow direct comparison? As I said before, I think this is an interesting manuscript, but I really struggle with the potential use for sea-level studies.

***Answer:*** *All the SLIP presented in Figure 11 are in close proximity (added in lines 526-527). The figure also presents the predicted RSL based on two GIA models, calculated specifically for the Dalmatian coast, indicating low isostatic contribution. Bechor et al. (2020) specified the calculated GIA rate on the Dalmatian coast as between -0.17 to -0.22 mm/a in their Table 3 (information added in lines 555-556).*

That said, it really reminds me of works done by Cearreta and collaborators in the North of Spain in reclaimed coastal areas. I think the authors could refocus the manuscript that way. If they do so, I think that the micropaleontological and geochemical analysis would need to be a bit more detailed. I also think that the disagreement between the historical data and the older OSL sample need to be addressed more in depth. Considering that this is one single sample, it could be considered what could have affected the result, such as sediment mixing.

I am sorry that I am not being more supportive and I hope that the authors can revisit the manuscript and get the research published.

***Answer:*** *Dating the saltwork****-****operation* *period based on historical archives, cannot provide a continuous record for the considered period since it relies on documents that describe episodic information. One of the main achievements of the current study methodology is the ability to recognize the saltwork unit and to date by OSL the beginning and the end of this period. This contribution is stated also by Reviewer 3: "I’ve also appreciated the use of OSL dating, which proved to be an efficient chronological method within these depositional contexts, deprived of organic remains".*

**Reviewer #2:** This is a very interesting manuscript, but despite the abundance of data it does not provide a conclusive interpretation. It is a manuscript based on sedimentological and paleontological (foraminifera and ostracods) description of three sediment cores. Unfortunately, the discussion is not based on interpretations of the data obtained from the cores.

Therefore, the foraminifera, geochemical data, and ostracods are an important source of information that need more attention and better interpolation in the text. The interpretation of the conditions in the saltpans is speculative.

***Answer:*** *We agree, more detailed interpretation has been added to the Discussion chapter.*

The whole story is based on representatives of the genus Ammonia. For some species, such as A. veneta or A. parkinsoniana, the fact that they are euryhaline, for authors, is very important and has been emphasized several times to obtain more saline conditions. But euryhaline species in themselves are characterized by covering a wide range of salinity. To be consistent with their interpretation, they referred to work in which species were abundant in saline environments and failed to say anything about ammonias from the eastern Adriatic and their distribution in brackish water (the paragraph needs to be better referenced).

Why were changes in foraminifera composition not used to show changes in saline areas (Supplementary data 1)? There is no biodiversity or ecological indices (studying of aliquots of 50 specimens?) to suggest that conditions have become more severe.

Why did the authors use the term dead foraminifera? What types of foraminifera can we find in historical material?

The same problem that data from ostracod assemblages are not considered.

***Answer****: We thank the reviewer for this list of questions and suggestions. We agree indeed, with the* *reviewer's statement: "Euryhaline species in themselves are characterized by covering a wide* *range of salinity". In our dataset, we found only one foraminifera representative of this wide-range salinity: A. veneta. As discussed in the manuscript, our interpretations rely on this species' occurrences dominating the assemblage (samples with relatively high dominance values) parallel to the decrease of marine taxa mirroring the ostracod data. Ammonia parkinsoniana is not euryhaline. It mostly lives abundantly in shallow subtidal sand in moderately sheltered normal marine salinity environments. However, occasionally it lives in hyposaline water (10-37 psu), for example in the northern Adriatic (Jorissen 1988). We added such information in the supplementary data 1. Indeed, the samples in our datasets are composed of relatively low abundances of microorganisms, as expected, in marginal marine environments. Nonetheless, as stated in the Material and methods section, studying samples with a quantity of 50 shells has already proven as a sufficient number needed for a relatively robust environmental interpretation (Kemp et al., 2020). The reviewer is right concerning the incorrect usage of the term "dead foraminifera" We corrected it in the revised manuscript.*

The geochemical data as tracers for identification of a terrestrial: line 455 and man made reddish layer and line 471… potentially resulted from anthropogenic activities…

***Answer:*** *"Man made reddish layer" is a wrong statement, the revised manuscript describes the process of natural terra rossa that has been brought to pave the saltpans****.***

Specific remarks

Brunovic et al. have studied cores from the island of Cres (northern Adriatic not Dalmatian coast).

***Answer:*** *Agree, the manuscript has been changed accordingly.*

WORMS for taxonomic identification, and not Cimerman & Langer?

***Answer:*** *Although WoRMS, which we refer to in our manuscript, includes all up-to-*

*date taxonomic information regarding species used in our study, we accepted the editor's request, and added, a taxonomic reference list of the most common species mentioned in the text in the revised supplementary data 1.*

**Reviewer #3:** The manuscript is very interesting in approach and it furnishes multi-proxy data (sedimentological, paleontological and geochemical data) from substantially unexplored, Mediterranean sedimentary successions: man-made intertidal saltpans. I’ve also appreciated the use of OSL dating, which proved to be an efficient chronological method within these depositional contexts, deprived of organic remains.

The Authors highlight the novelty of this study mainly in terms of RSL reconstructions. I substantially agree, however I think that paleoenvironmental interpretations (upon which RSL data are based) needs to be improved.

My major comments are as follows:

• A solid paleoenvironmental interpretation of core units is necessary. Each unit is described (4. Results) but not interpreted, with the exception of the paved layer and the so called “euryhaline interval” (first part of the discussion). I suggest to better discuss all the paleoenvironmental phases identified within the studied succession (5.1.) before presenting the RSL inferences (5.2.): pre-saltworks interval; saltworks interval (including the paved layer and the “euryhaline layer”) and post-abandonment interval highlighting key features and differences.

***Answer:*** *Agree, the Discussion chapter has been revised accordingly*.

• The Authors stated that they used salinity ecological groups, however in the main text they reported “coastal/lagoonal taxa” about foraminifers (e.g., line 311) and they referred to coastal taxa in Figures 4, 7. I think that this is confusing and the merging of different salinity groups determines a loss of information. Moreover, I wonder which is the meaning of the taxa highlighted in green in the supplementary data 1.

**•** The counted shells/valves are generally low, I suggest to take into consideration/discuss this issue and to highlight the smallest samples (<50).

***Answer:*** *We agree with the reviewer and now have used the same salinity ecological groups along the text and the figures. We do not think that there is a loss of information while merging species having similar known ecological, i.e., salinity preferences. All census data are available in supplementary data 1 & 2. The representation of assemblage data is appropriate to our research questions, as done in many ecological studies. The taxa are not highlighted in green anymore in supplementary data 1.*

**•** Lines 517-518: from which core sample the OSL age of 1215±175 CE has been obtained? This RSL index point is not clear to me.

***Answer:*** *The OSL age of 1215±175 CE is the mean* *value between the time when the salt production was initiated, dated by OSL to 1040±50 CE, and the date when the salt production ceased, dated by OSL to 1390±30 CE (lines 513-518). Additional lines have been added to the revised manuscript explaining the index point (lines 536-541).*

Yours sincerely,

Benny Bechor (corresponding author) on behalf of all co-authors

Department of Maritime Civilizations,

School of Archaeology and Maritime Cultures

University of Haifa,

Haifa 31905, Israel

E-mail: bbechor1@gmail.com

+972 52 6093437
